# Large language models with retrieval-augmented generation enhance expert modelling of Bayesian network for clinical decision support

**DOI:** 10.1007/s11548-025-03524-9

**Published:** 2025-11-03

**Authors:** Mario A. Cypko, Muhammad Agus Salim, Aditya Kumar, Leonard Berliner, Andreas Dietz, Matthaeus Stoehr, Oliver Amft

**Affiliations:** 1 Hahn-Schickard-Gesellschaft für angewandte Forschung e.V., 79110 Freiburg, Germany; 2 Intelligent Embedded Systems Lab, University of Freiburg, 79110 Freiburg, Germany; 3 Department of Radiology, Staten Island University Hospital – Northwell Health, NY 10305 Staten Island, USA; 4 Department of Otolaryngology, Head and Neck Surgery, University Hospital Leipzig, 04103 Leipzig, Germany

**Keywords:** Retrieval-augmented generation, Bayesian networks, Knowledge engineering, Clinical decision support systems, Large language models, Causal modelling in healthcare

## Abstract

****Purpose**:**

Bayesian networks (BNs) are valuable for clinical decision support due to their transparency and interpretability. However, BN modelling requires considerable manual effort. This study explores how integrating large language models (LLMs) with retrieval-augmented generation (RAG) can improve BN modelling by increasing efficiency, reducing cognitive workload, and ensuring accuracy.

****Methods**:**

We developed a web-based BN modelling service that integrates an LLM-RAG pipeline. A fine-tuned GTE-Large embedding model was employed for knowledge retrieval, optimised through recursive chunking and query expansion. To ensure accurate BN suggestions, we defined a causal structure for medical idioms by unifying existing BN frameworks. GPT-4 and Mixtral 8x7B were used to handle complex data interpretation and to generate modelling suggestions, respectively. A user study with four clinicians assessed usability, retrieval accuracy, and cognitive workload using NASA-TLX. The study demonstrated the system’s potential for efficient and clinically relevant BN modelling.

****Results**:**

The RAG pipeline improved retrieval accuracy and answer relevance. Recursive chunking with the fine-tuned embedding model GTE-Large achieved the highest retrieval accuracy score (0.9). Query expansion and Hyde optimisation enhanced retrieval accuracy for semantic chunking (0.75 to 0.85). Responses maintained high faithfulness ($\ge$0.9). However, the LLM occasionally failed to adhere to predefined causal structures and medical idioms. All clinicians, regardless of BN experience, created comprehensive models within one hour. Experienced clinicians produced more complex models, but occasionally introduced causality errors, while less experienced users adhered more accurately to predefined structures. The tool reduced cognitive workload (2/7 NASA-TLX) and was described as intuitive, although workflow interruptions and minor technical issues highlighted areas for improvement.

****Conclusion**:**

Integrating LLM-RAG into BN modelling enhances efficiency and accuracy. Future work may focus on automated preprocessing, refinements of the user interface, and extending the RAG pipeline with validation steps and external biomedical sources. Generative AI holds promise for expert-driven knowledge modelling.

## Introduction

Modern medicine is shifting from static, population-based guidelines to a patient-specific approach called model-guided medicine [1]. Model-guided medicine leverages computational models to integrate patient data, clinical guidelines, and contextual factors into adaptable representations that guide decision-making. Unlike traditional evidence-based medicine, model-guided medicine continuously refines models based on real-time patient data, ensuring treatment strategies remain adaptive and transparent [2]. At the core of model-guided medicine is the ability to structure comprehensive and distributed medical knowledge into comprehensible and interpretable frameworks. Probabilistic graphical models, particularly Bayesian networks (BNs) by Pearl [3], are well-suited for this purpose. BNs represent causal relationships between medical variables and enable probabilistic inference, allowing clinicians to mathematically assess diagnostic and prognostic uncertainties, which is critical for patient safety and compliance in critical healthcare situations [4]. By incorporating expert knowledge, BNs facilitate structured decision support in domains such as oncology, cardiology, and intensive care [5]. While BNs provide key advantages, their design remains a highly labour-intensive task that requires extensive domain expertise with a time-consuming encoding of knowledge into a BN model. Current approaches, including data-driven and expert-driven methods, face scalability issues due to the significant manual effort involved [6]. Recent advances in large language models (LLMs) and Retriever Augmented Generation (RAG) offer new opportunities to extract structured knowledge from medical texts and encode in BN.

We propose an LLM-RAG framework for semi-automatic BN modelling. Our methodology combines expert-curated causal structures with LLMs and external knowledge retrieval to improve accuracy and consistency in BN generation. The proposed framework was evaluated in a case study on lymph node staging in laryngeal cancer. The key contributions of this study are:

1. We developed an LLM-RAG pipeline for BN expert modelling to reduce the manual input needed for BN design, while preserving interpretability.
2. We defined a causal structure of medical idioms that provides a meta-schema for automated and manual BN construction.
3. We evaluated the LLM-RAG-supported BN expert modelling in a clinical setting, demonstrating improved efficiency and relevance, while emphasising the importance of expert curation.

## Related work

### Modelling Bayesian networks

BN modelling remains labour- and time-intensive, requiring collaboration between domain experts and knowledge engineers [7]. Data-driven methods such as the Peter-Clark (PC) algorithm infer causal structures from datasets, if sufficiently data are available, but often fail to capture clinically relevant causal relationships [8]. Tools like GeNIe and the SMILE engine incorporate prior domain knowledge to improve the algorithms interpretability [9], but with comprehensive models and limited data, the modelling work remains with the domain expert [6].

Expert modelling typically involves collaboration between domain experts and knowledge engineers. Domain experts extract knowledge from medical sources, while knowledge engineers encode this knowledge into a causal structure within a comprehensive knowledge graph. For example, a simplified model with 94 variables may require 40 h of teamwork, while a more complex model with over 1000 variables can take two to three years [10, 11].

### Large language models-based modelling of causal knowledge

Recent advancements in LLMs, including Llama 2, BioLLaMA-3, GPT$-$3.5, GPT-4, Gemini Pro, BioBERT, and BioLLaMA-3, have shown promise in causal knowledge modelling [12–14]. Prompt engineering, such as creating more precise, context-rich, and structured prompts, enables LLMs to produce more relevant and accurate outputs. Carefully constructed prompts enhance causal reasoning by guiding the model towards better understand complex relationships. However, LLMs still struggle with temporal causality and counterfactuals, often falling into the post hoc fallacy [15].

### Retrieval-augmented generation for structured clinical modelling

RAG enhances LLMs with external knowledge sources to improve factual accuracy and reduce hallucinations [16]. RAG techniques include, for example, query expansion, HyDe-based augmentation, and predefined prompts. Embedding models (LLM-based) are used as a basis for external knowledge retrieval. Despite their promising features, RAG models face challenges in data matching and contextual consistency and are prone to generating irrelevant information if the retrieval process is misaligned [17]. The choice of a suitable embedding model and additional fine-tuning methods is therefore essential for the overall performance of an RAG. To our knowledge, no studies have yet combined LLMs and RAG with Bayesian networks for causal modelling.

## Methods

**Fig. 1:**
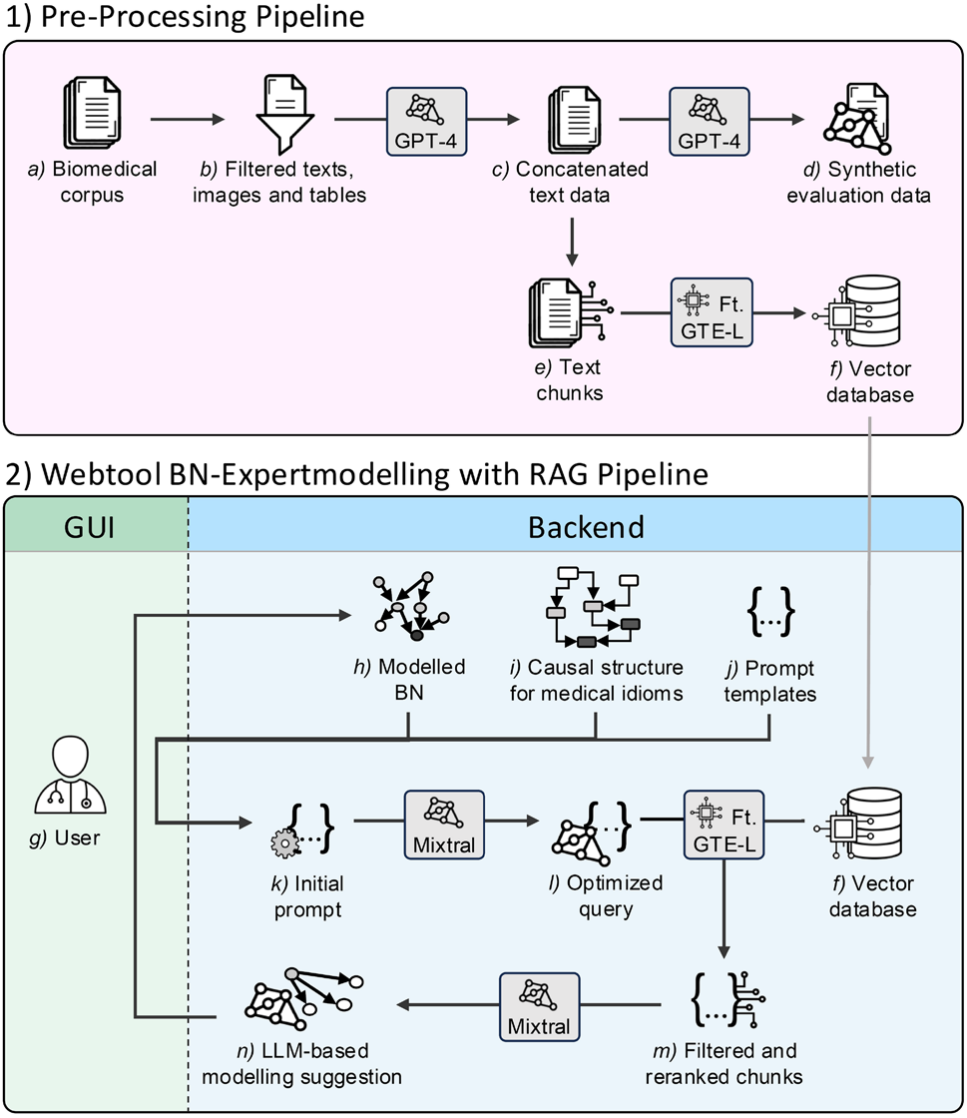
Overview of our methodological approach for the LLM-RAG enhanced web-based BN modelling tool. Our approach includes (1) pre-retrieval processing pipeline that is executed only once and (2) the web-based BN modelling tool with integrated RAG pipeline that is further divided into graphical user interface and backend

Our approach integrated LLM-supported generation of modelling proposals with expert-based curation. The methodology utilised two LLM-RAG processing pipelines as shown in Fig. 1: *1)* a pipeline for pre-retrieval processing of medical knowledge and *2)* a BN modelling web tool with an RAG pipeline. To provide the RAG retrieval contextual information about BNs in the medical field, we first unified two medical BN frameworks into a *i) causal structure for medical idioms*. To ensure robust and contextually accurate BN modelling, our system integrated two LLMs (GPT-4 [18] and Mixtral 8x7B [19]) and one LLM-based embedding model (fine-tuned GTE-Large) with distinct roles at different stages of the pipeline. To assess the effectiveness of the RAG knowledge retrieval and generated modelling proposals, we evaluated both the retrieval performance of embedding models and the usability of the system with clinicians. Selecting a suitable embedding model was crucial for our pipeline, and an evaluation is detailed in the results.

### Causal structure for medical idioms

**Fig. 2:**
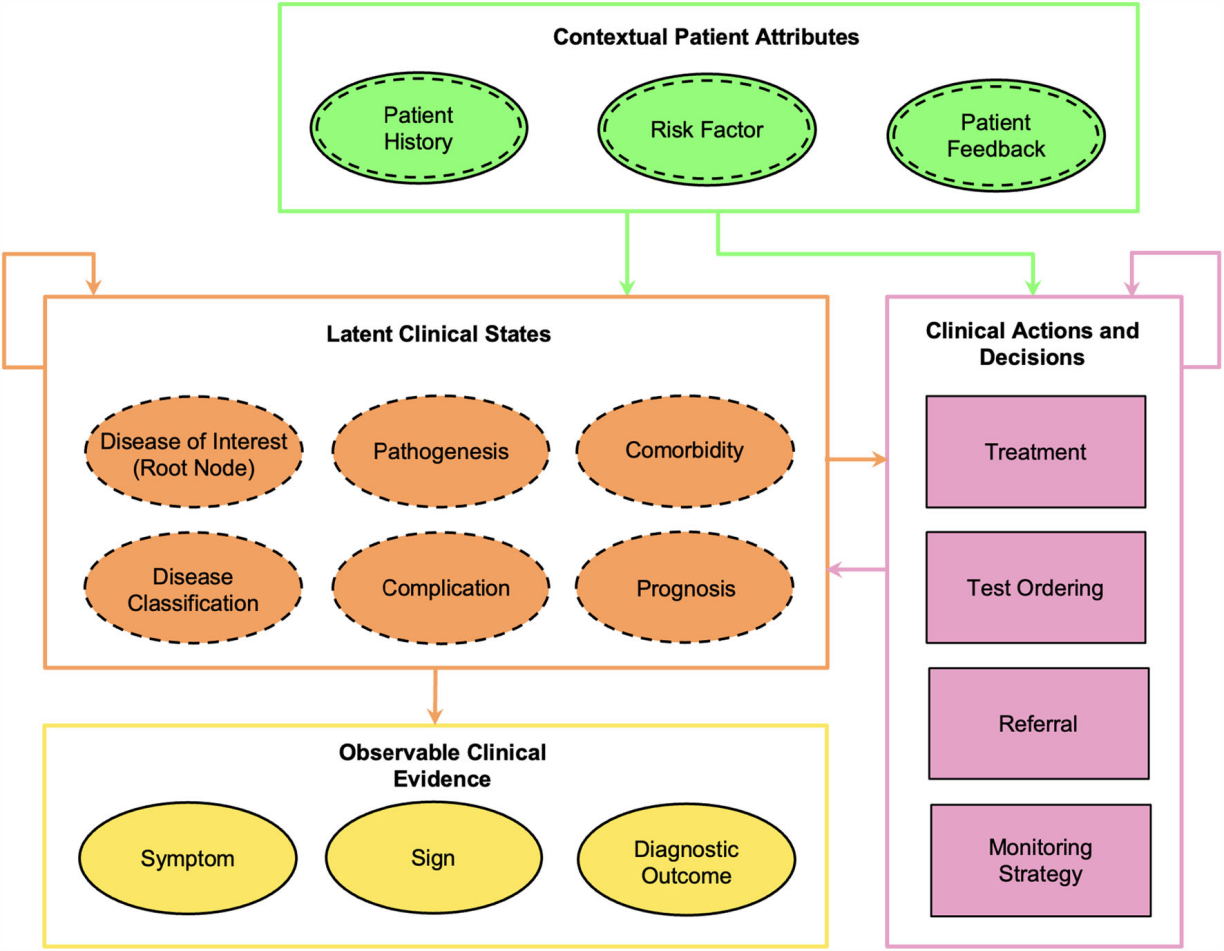
Structured causal model for clinical idioms with semantically encoded node types. The diagram distinguishes four node classes: (1) manually set patient attributes (green ellipses, solid outer, and dashed inner border), (2) latent clinical states (orange ellipses, dashed border), (3) measured clinical evidence (yellow ellipses, solid border), and (4) decision/action nodes (magenta rectangles). Based on Cypko et al. [11] and Kyrimi et al. [20], the structure supports interpretable BN generation by constraining the causal dependencies between node categories. Intra-class causal links are omitted for clarity

To improve the interpretability and structure of BNs for medical applications, we developed an optimised causal structure for medical idioms, integrating previous frameworks from Cypko et al. [11] and Kyrimi et al. [20], see Fig. 2. The meta-structure from [11] defines syntactic modelling rules regarding the observability and connectivity of variable types, such as patient condition, examinations, and therapy decisions, to ensure structural consistency in large-scale models. In contrast, the framework from [20] describes semantic medical idioms and their conceptual relations, primarily to facilitate knowledge categorisation and model reuse. The resulting causal structure in Fig. 2 combines both frameworks and defines four semantically distinct types of variables. Each type differs in whether they are observable, measurable, manually set, or inferred:

1. *Context variables*, such as age or lifestyle factors, are observable but not measured and are manually set by the user;
2. *Latent variables* represent internal clinical states that are not directly observable and must be inferred from other information;
3. *Evidence variables* are observable and objectively measured, typically found at the leaves of the network (e.g. symptoms or test results);
4. *Decision variables* represent clinical choices or actions and are set subjectively by the user.

Explicit categorisation of variable types enables systematic modelling and defines allowable causal relationships. The resulting structure supports scalable and consistent development of interpretable BNs for clinical decision support.

### Pre-retrieval processing

The pre-retrieval processing stage refines textual data before retrieval. This step optimises search relevance and efficiency by segmenting documents, extracting information from flowcharts and tables, removing irrelevant content, and transforming text chunks into dense vector representations.

In our pipeline, we first *a)* created a biomedical corpus with domain-specific medical knowledge. For our study, we compiled knowledge from medical guidelines and textbooks on laryngeal cancer (cf. [21–23]). To *b)* remove irrelevant text and extract flowcharts and tables from the corpus, we applied regex filtering [24] and umPyMuPDF4LLM API [25], respectively. GPT-4 then interpreted the extracted information, flowcharts, and tables and combined them into a *c)* concatenated text. Due to its high computational cost, GPT-4 is impractical for frequent interactions within our pipeline, but its high performance in language processing makes it well-suited for general LLM tasks [18]. The concatenated text was used as the foundation for creating both a *d) synthetic evaluation dataset* to fine-tune embedding models and a *f) vector database*, which acts as the knowledge base for the RAG. The synthetic evaluation dataset was generated again with GPT-4 using prompts of various complexity, which we adapted from a prompt list by the RAGAS framework [26]. We generated a total of 2,070 questions, from which we randomly selected 30 moderately difficult and 30 difficult questions. We then used the synthetic dataset to fine-tune embedding models using [27] and identified our *fine-tuned GTE-Large* as the most suitable model for our RAG pipeline. The vector database required additional *e)* chunking steps by combining *recursive chunking* [28], *semantic chunking* [29], and *propositional chunking* [30], to extract words, sentences, and paragraphs, which were then semantically combined and encoded in *f)* a vector space using the fine-tuned embedding model [31].

### Retrieval-augmented generation pipeline

Our system used a specialised RAG pipeline to retrieve domain-specific information and generate relevant prompts. For BN modelling, *g)* the user had access to *h)* the previously modelled BN model (if already available), *i* the causal structure for medical idioms, and *j)* adopted prompt templates from the RAGAS evaluation framework [26]. Information was dynamically presented based on the user’s interaction with the system. Based on the user’s interaction and desired modelling, the system assembled *k)* an initial prompt. The initial prompt was *l)* optimised using the Mixtral LLM and *(p)* query optimisation techniques, such as Query Expansion [32] and HyDe-based augmentation [26], to improve the relevance and clarity of the context. Mixtral 8x7B was used due to its ability to handle over 30k tokens (i.e. sets of words) efficiently [19]. The optimised query was then used by the fine-tuned embedding model to retrieve top 10 chunks from the *f)* vector database and then *m))* reranked and filter the most relevant 5 chunks. Finally, the Mixtral LLM *n)* generated a set of BN modelling suggestions. Users could either accept the generated suggestions to extend or adapt the *h)* BN model or refine their request to generate new suggestions, enabling continuous and flexible modelling within the tool.

### Web-based BN modelling tool

**Fig. 3:**
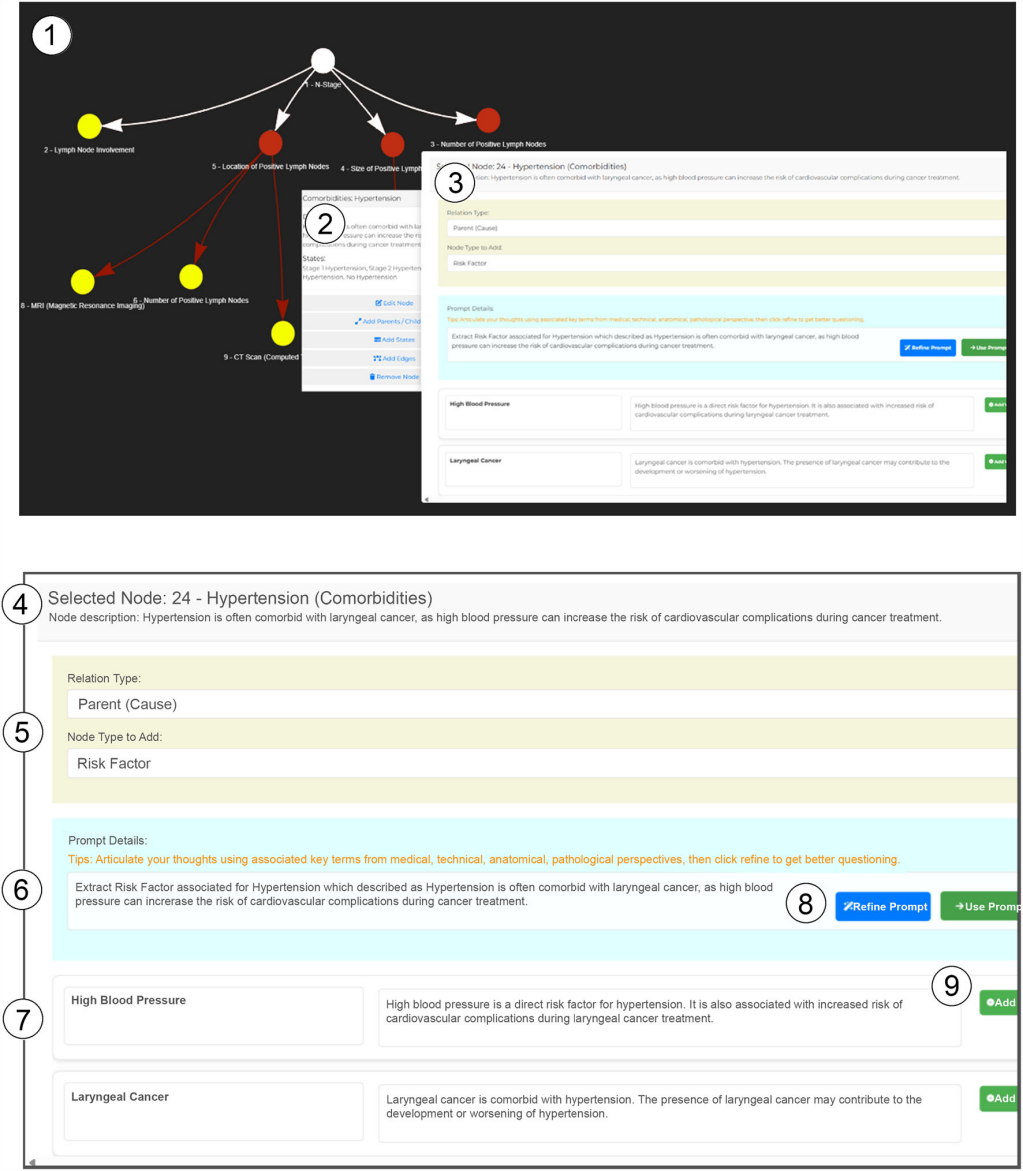
Screenshots of the web-based BN modelling tool. The BN modelling tool can support clinicians in an exemplary 6-step workflow assisted by LLM-based suggestions (shown in the panel on the right)

The developed web-based modelling tool provides an interactive environment for domain experts to efficiently construct BNs. As shown in Fig. 3 (top), the interface consists of *1)* the model viewer, *2)* a menu panel, and *3)* an LLM-modelling panel. The model viewer allows users to view and rearrange nodes, as well as open the menu panel by clicking on nodes or edges. The menu panel offers several creation and editing functions specific to the selected node or edge. Except for renaming or deleting, each function opens the LLM-modelling panel. The LLM-modelling panel, shown in Fig. 3 (bottom), includes *4)* a header with node or edge specifications, *6)* a prompting field, and *7)* an LLM-answer field. For example, when adding nodes to "hypertension", see  *4)*, the panel also includes *5)* a medical idioms field, where users select the relationship type (parent, child, or sibling) and the relevant medical idiom. The selected idiom and relationship type, together with the node or edge context, form the input used to generate *6)* an initial prompt. The user can either *8)* use the generated prompt directly or refine it by editing the content or selecting *‘Refine Prompt’* for alternative suggestions. Once accepted, the tool presents LLM-based answers, see *(7)*, each with associated reasoning. Users can *9)* add suggested nodes by clicking the *‘Add’* button, and the nodes are automatically placed in the model and visible after closing the panel. The visible actions for the user are therefore limited to selecting the desired modelling, adjusting the modelling accuracy, and selecting the modelling suggestions.

### Fine-tuned embedding models

We evaluated the embedding models BGE, PubMedBERT, and a fine-tuned variant of GTE-Large in combination with three chunking strategies: semantic, propositional, and recursive. To assess performance, we focused on three key metrics: retrieval accuracy, answer relevance, and faithfulness. Retrieval accuracy was measured using the Hit Rate @5, which determines how often the relevant medical information appeared among the top five retrieved results. The evaluation considered the effects of different embedding models, chunking strategies, and query expansion techniques on retrieval performance. Answer relevance was assessed by comparing the retrieved documents to the clinician’s query using a cross-encoder re-ranking approach. The re-ranking selected documents that matched the query’s intent and ensured contextual consistency of the results. Faithfulness score measured how factually accurate and well-grounded the generated responses were in relation to the retrieved sources. A verification pipeline compared the generated answers to the original medical guidelines to determine the level of factual consistency.

### User study

We conducted a user study with 4 clinicians, who were domain experts in head and neck cancer staging. Clinicians were asked to construct a BN for N-staging in laryngeal cancer using the web tool. The study aimed to assess the ease of use, task performance, and the system’s ability to maintain the correct causal dependencies, decision node structure, and the consistency of expert annotations with LLM-suggested refinements.

The study took place in individual online meetings, each lasting one hour. The first 10 min were spent familiarising the clinicians with the tool via video tutorials, and the remaining 50 min were dedicated to modelling. Clinicians were encouraged to think aloud, share their screen, and ask questions throughout the process. The study manager ensured the tool’s functionality and intervened minimally, only assisting when the clinician encountered difficulties.

We measured task completion time, retrieval accuracy, user satisfaction, and cognitive workload using the NASA Task Load Index (NASA-TLX) [33]. Additionally, clinicians provided feedback on their experiences with cancer staging, BN modelling, and chatbots.

## Results

### Retrieval-augmented generation pipeline

The performance of the RAG pipeline was systematically evaluated to understand its effectiveness in enhancing retrieval accuracy and answer relevancy for BN modelling. The evaluation focused on two critical aspects: the performance of different embedding models and the quality of responses generated using a synthetic evaluation dataset. The analysis identified key strengths and limitations of the pipeline and provided insights into its optimisation and practical applicability.

#### Embedding models performance

Our heatmap analysis, as illustrated in 4, revealed four key findings. First, recursive chunking outperformed other methods, with GTE-Large achieving a baseline score of 0.88 and improving to 0.90 after fine-tuning. Semantic chunking (best performing with BGE-Large) ranged from 0.75 to 0.85, while propositional chunking scored lowest (0.47 to 0.62). Second, retrieval performance improved with techniques like query expansion (0.75 to 0.82) and Hyde optimisation (0.75 to 0.85); however, fine-tuning slightly reduced the improvement achieved through Hyde optimisation, lowering the score from 0.85 to 0.80. Third, generated responses showed high faithfulness across models, all scoring above 0.9, with propositional chunking achieving 0.97. Fourth, RAG pipelines enhanced answer relevancy from 0.73 to 0.82, with a 0.79 correlation between retrieval hit rate and relevancy.

**Fig. 4:**
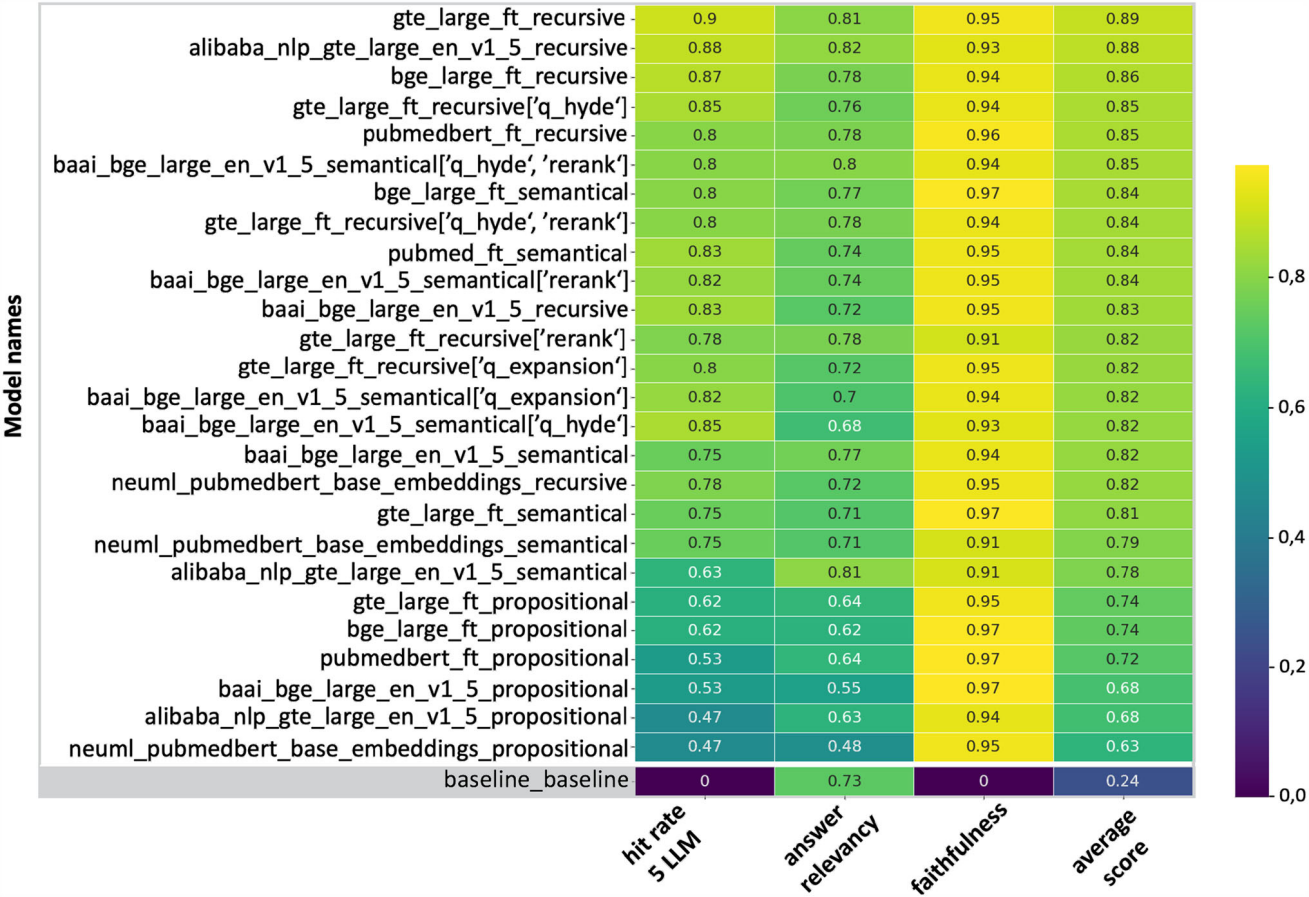
Heatmap of the effectiveness of different chunking methods in the RAG pipeline. Recursive chunking consistently outperformed alternative strategies

#### Synthetic question evaluation

To assess retrieval performance and answer relevancy, we analysed questions from the synthetic evaluation dataset. The focus was on the GTE-baseline and its fine-tuned version, which achieved the highest scores. A targeted evaluation identified insightful cases by examining queries, associated scores, retrieved contexts, and the corresponding reasoning provided by the LLM.

Our analysis revealed three key findings. Firstly, structural inconsistencies in LLM responses occasionally caused parsing challenges and metric errors. Examples include extra characters (e.g. brackets that interfered with parsing), deviations from expected phrasing, excessive JSON outputs, and rare instances of providing information not found in the retrieved context. Despite structural inconsistencies, the Mixtral 8x7b model adhered strongly to prompts. Secondly, citation errors occurred, such as using incorrect chunk IDs or referencing the array index instead of the actual chunk. For example, the LLM cited [citation: 0] instead of [192] or referenced retrieved context like [jugular lymph nodes (level III)] instead of the correct ID [541]. Lastly, while the LLM consistently generated responses faithful to the retrieved context, it occasionally failed to comply with instructions for brevity. Instead of concise answers, it produced unnecessarily long responses, which, although accurate and contextually relevant, could reduce efficiency in practical applications.

### User study

#### N-stage modelling

**Fig. 5:**
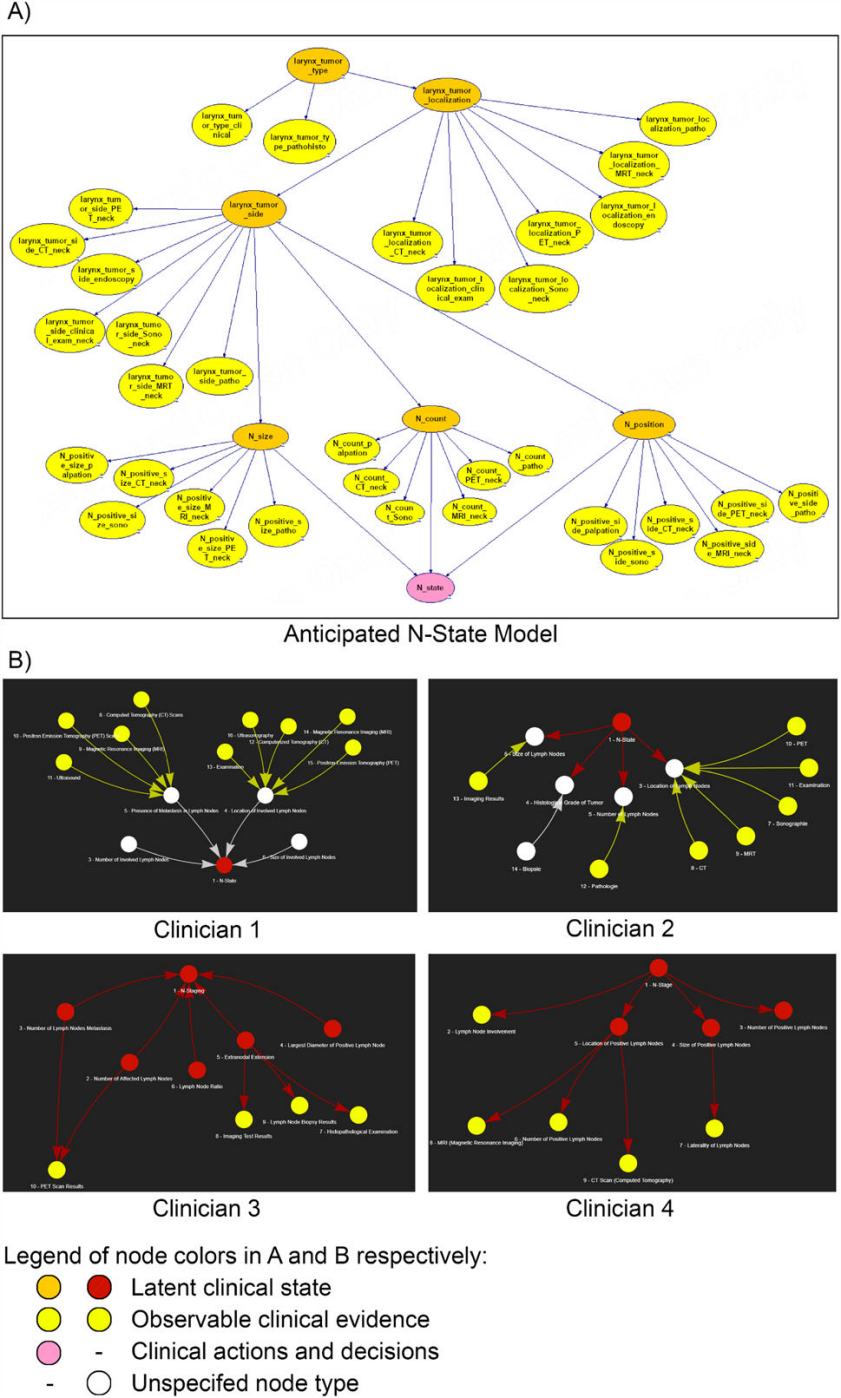
A) Anticipated N-state model as shown in GeNIe 3.0 [9]; B) modelling structures of the four clinicians within the LLM-RAG modelling tool. Colours represent variable types as presented in the figure legend and in Fig. 2

The results of the N-stage modelling by the four clinicians show varying degrees of alignment with the anticipated N-stage model from the TreLynCa model [11], see Fig. 5.

Each clinician approached the modelling differently. The two more experienced clinicians produced the highest node counts (13 and 14 nodes). The two clinicians without prior BN modelling experience and less clinical experience created less complex models (9 and 10 nodes). All clinicians included information for the N-stage, such as lymph node size, number, location, presence of metastasis, and diagnostic methods like imaging, biopsy, CT, PET, and MRI. With the limited working time of 60 min, the resulting BN models varied in their completeness and focus.

However, the most comprehensive model included incorrect selections of medical idioms and invalid causal dependencies. Such modelling errors arose from two main sources: the LLM occasionally deviated from the predefined structures and from experienced clinicians showing confidence in adapting LLM suggestions through manual adjustments, but also introducing causality errors. Interestingly, the less experienced clinicians rarely made adjustments to the LLM suggestions and their models correctly applied both the medical terms and the causal relationships in most cases.

#### Usability measurements

**Fig. 6:**
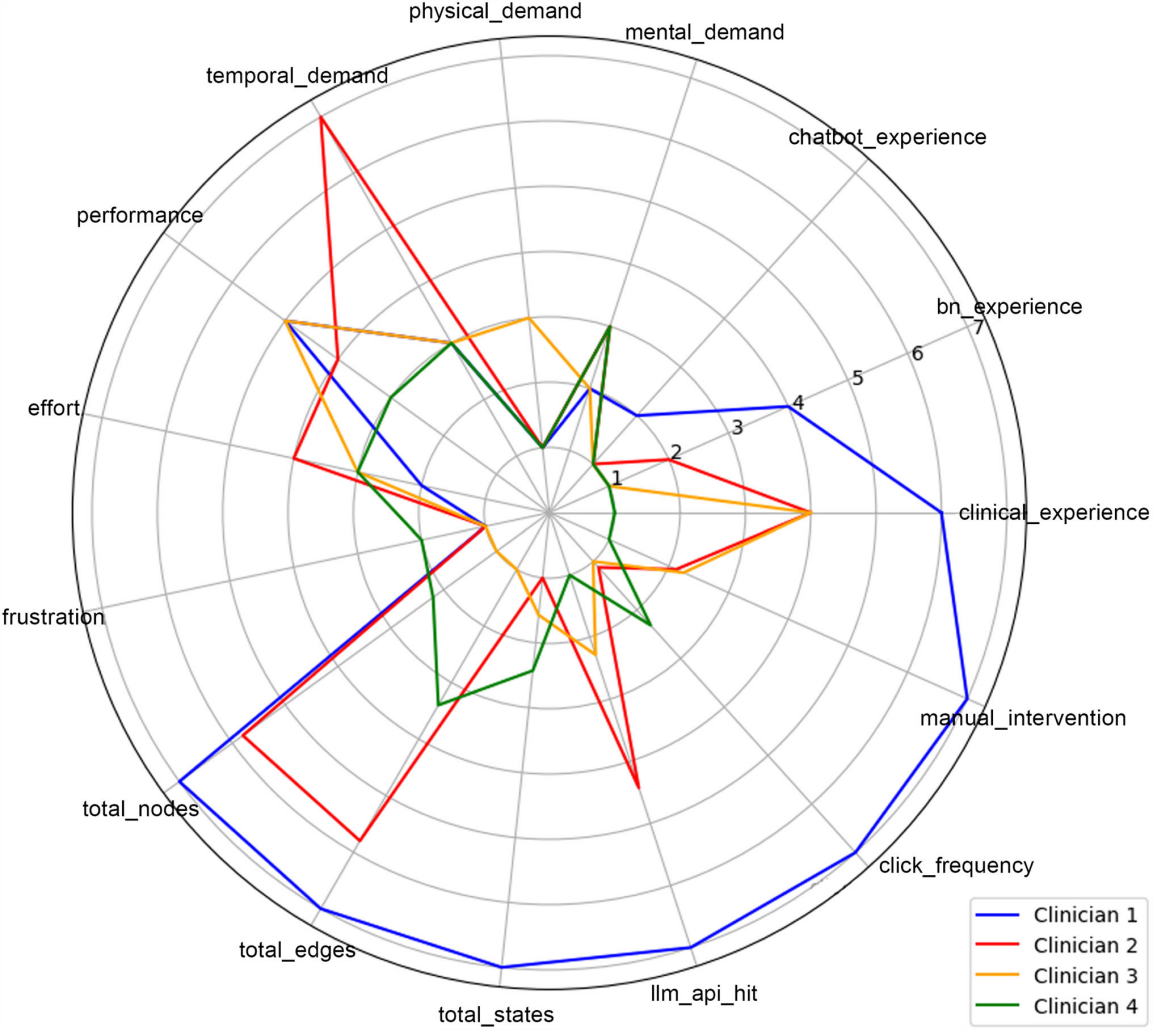
Radar chart of usability metrics for four clinicians who used the BN modelling tool. Evaluation included cognitive workload (e.g. mental and temporal demand) based on NASA-TLX, interaction frequency (e.g. click frequency, manual intervention), modelling activity (e.g. total nodes, edges, and states), and prior experience (e.g. clinical expertise, BN modelling, and chatbot familiarity). Results from interaction frequency and modelling activity were normalised to a 7-point scale, ranging from the lowest to the highest recorded values for better comparability

The evaluation study provided first insights into clinician interactions with the LLM-assisted BN modelling tool, see radar chart in fig. 6. Clinician 1, with the highest BN and clinical experience, showed the best performance, though with high manual interventions, indicating a strong understanding of the tool but frequent need for adjustments. Clinician 2, with slightly less BN experience and clinical experience, performed well but required more effort and showed a moderate level of manual interventions. Clinician 3 had similar performance to Clinician 1 but demonstrated significantly lower BN experience, which resulted in higher effort during tool use. Nevertheless, the user exhibited low frustration and maintained overall efficiency. Clinician 4, with the lowest BN and clinical experience, showed the weakest performance and required fewer manual interventions, indicating simpler interactions but reduced effectiveness in using the tool.

**Fig. 7:**
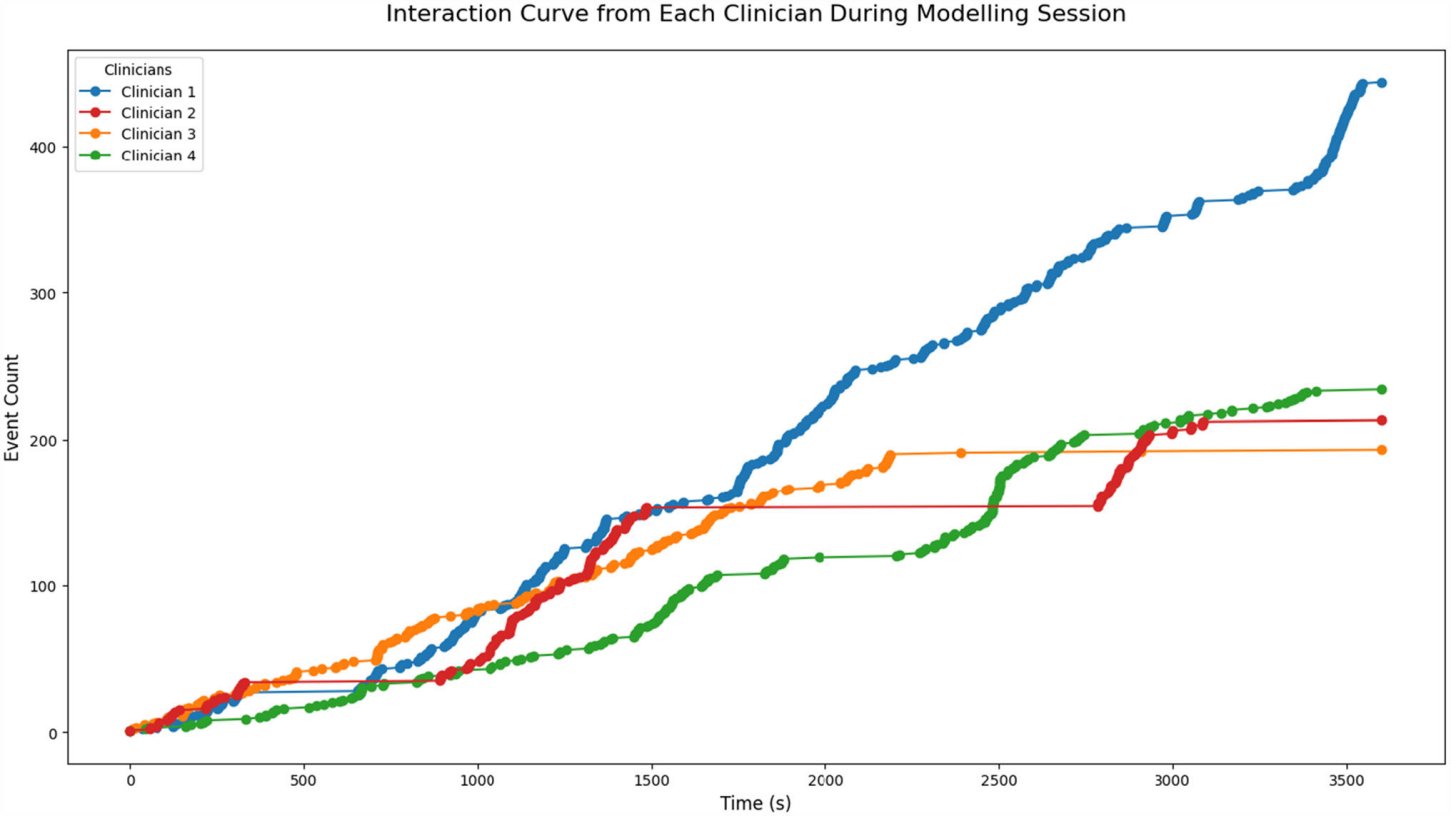
Interaction curves of all clinicians during the 60-minute modelling session. The y-axis represents the cumulative number of user events (e.g. edits, prompts, confirmations), while the x-axis shows the elapsed time in seconds. Flat segments indicate interruptions (e.g. clinical calls), with Clinician 2 and 3 experiencing prolonged pauses of up to 20 min, and Clinician 4 showing frequent shorter disruptions. Steeper slopes reflect higher interaction intensity

Clinician 2 and 3 faced up to 20 min interruptions due to clinical calls, which caused delays and required time to reorient when returning, see Fig. 7. Clinician 4 experienced frequent shorter disruptions, also affecting workflow continuity. Since all disruptions occurred within the 60-minute sessions, the modelling time was shorter for the affected clinicians.

#### Qualitative feedback

The majority of clinicians described our tool as "intuitive and quick" and the LLM suggestions as helpful in structuring dependencies. Despite the strengths, some challenges were identified. Clinicians reported that understanding the hierarchy of decision nodes required additional learning time, particularly for those with less prior experience in BN modelling. Additionally, minor technical issues, such as browser compatibility concerns, were reported.

## Discussion

This study demonstrates the potential of integrating LLMs with RAG for BN modelling, improving efficiency and reducing cognitive workload. From a methodological perspective, this work provides a significant contribution by exploring the possibilities of LLM-RAG-based support in expert BN modelling. However, several areas require further investigation.

One key challenge was the inconsistency in enforcing causal structures for medical idioms. Despite the system’s design to maintain structured clinical reasoning, errors arose from LLM misinterpretations and occasional failures to adhere to causal constraints. Future systems should integrate rule-based mechanisms to strictly enforce the predefined causal relationships between variables and extend the RAG pipeline with approaches like LLM-as-a-judge to evaluate retrieved chunks before generating BN modelling suggestions [34, 35]. Additionally, incorporating external sources such as PubMed abstracts could address gaps in the biomedical corpus when handling complex queries.

Practical challenges, such as clinical interruptions, also impacted modelling efficiency. The system should include features like recalling the last modification or providing context-sensitive information boxes to support users during disruptions.

Further methodological research is essential to expand the scope and applicability of LLM-RAG-based support in expert BN modelling. First, evaluating embedding models on diverse datasets could determine whether GTE-Large is consistently suitable for medical domains. Second, the automation of the pre-retrieval pipeline, including the fine-tuning of embedding models, and the integration of both into the web tool would allow users from different medical domains to upload relevant documents and initiate modelling workflows autonomously. Third, exploring alternative LLMs could identify cost-effective replacements for GPT-4 without compromising performance.

The integration of large language models with LLM-RAG bridges the gap between clinical reasoning and the causal dependencies required for building BNs. However, future efforts should aim to establish an optimal level of cognitive engagement rather than minimising cognitive load entirely. TNM served as a useful proof-of-concept due to its validated and comprehensive structure [11], but future models should incorporate molecular insights into tumour diseases to enable novel treatments and support multidisciplinary tumour boards.

The presented evaluation contributes to the broader discussion on the role of AI in clinical decision support. Transparent, reproducible knowledge representations, such as causal BNs, and expert-informed modelling workflows provide a robust foundation for trustworthy clinical AI [2, 36]. Ongoing development of the modelling tool focuses on integrating rule-based guidance, improving RAG performance, and enhancing the graphical user interface, with broader accessibility planned via https://bayesmedical.net.

## Conclusion

This study explored the integration of LLMs with RAG to support clinicians in BN modelling. The results demonstrate that AI-assisted modelling can enhance efficiency and reduce cognitive workload by streamlining knowledge retrieval and structuring. However, key challenges remain, particularly regarding the enforcement of predefined causal structures, the usability of AI-generated suggestions, and the need for better graphical user interface integration to indicate ongoing RAG processing.

Additionally, the study underscores the importance of balancing cognitive load in AI-assisted modelling. While automation can alleviate the burden of manual information retrieval and causal structuring, excessive reliance on AI-generated outputs may lead to errors or misinterpretations.

Beyond BN modelling, our findings contribute to a broader discussion on the role of generative AI in clinical decision support. Future hybrid approaches could integrate generative AI for initial structuring and as an indicator while relying on validated knowledge bases for reasoning, ensuring both transparency and clinical trust.
